# Genome sequence data of new *Paenarthrobacter nicotinovorans* FJ15 isolated from tobacco rhizosphere soil in Nanping, China

**DOI:** 10.1128/mra.00583-25

**Published:** 2026-04-08

**Authors:** Chao Ye, Wei Lin, Yong Lin, Jianyang Liu, Wei Li, Shiliang Hang, Jingchao Li, Xianlong Chen, Jing Wang

**Affiliations:** 1 Nanping Branch of Fujian Tobacco Company, Nanping, China; 2 Pucheng Branch of Nanping Tobacco Company, Pucheng, China; 3 Zhenghe Branch of Nanping Tobacco Company, Zhenghe, China; 4 Jianyang Branch of Nanping Tobacco Company, Jianyang, China; 5 Key Laboratory of Tobacco Pest Monitoring Controlling and Integrated Management, Tobacco Research Institute of Chinese Academy of Agricultural Sciences, Qingdao, China; University of California Riverside, Riverside, California, USA

**Keywords:** complete sequence, nicotine degradation

## Abstract

Here, we report the genome sequence of *Paenarthrobacter nicotinovorans* FJ15, which involves nicotine degradation isolated from soil in Nanping, China. The genome consists of a chromosome with 4,376,624 bp and a GC content of 63.15%, two plasmids size 274,256 bp and 95,657 bp.

## ANNOUNCEMENT

*Paenarthrobacter nicotinovorans* is a gram-positive soil bacterium renowned for degrading nicotine, whose catabolic genes are often located on a ~160 kb megaplasmid, pAO1 (1–4).

In this study, the nicotine-degrading (5) strain FJ15 (CGMCC 1.65031) was isolated from tobacco rhizosphere soil in Fujian, China. It degraded 82% of nicotine (1 g/L) within 20 hours (6). To elucidate its degradation metabolism, complete genome sequencing was performed.

Total DNA extraction of FJ15 was performed using the CTAB method. The complete genome sequence of FJ15 was determined using Pacific Biosciences (PacBio) Revio (with an insert size of 450 bp) and Illumina NovaSeq X Plus sequencers. A PacBio 10 kb sequencing library was prepared using the template Prep Kit 1.0 and sequenced with the Revio platform. An Illumina sequencing library was constructed using the TruSeq DNA PCR-free prep kit and sequenced with the NovaSeq platform. The raw reads were quality controlled as follows: Illumina reads were processed with fastp (v0.23.1), yielding 9,615,452 high-quality reads (1,449,685,158 bp), which provided approximately 305× coverage of the genome. The PacBio data consisted of 144,114 reads totaling 2,385,168,807 bp, with the longest being 44,578 bp, the shortest 1,321 bp, an N50 length of 17,267 bp, and an N90 length of 12,269 bp (see Table 1).

**TABLE 1:** *Paenarthrobacter nicotinovorans* FJ15 (CGMCC 1.65131) genomic features^*a*^

Genomic features		Number	
Genome project information	Genbank accession number	PX435363	
	Bioproject accession number	PRJNA1264549	
	Biosample accession number	SAMN48564267	
Illumina NovaSeq X Plus	Total number of high-quality reads	9,615,452	
	Total high-quality base pairs (bp)	1,449,685,158	
	Estimated genome coverage	305×	
PacBio (Chromosome)	Total sequence length (bp)	2,385,168,807	
	Total sequence number	144,114	
	Longest (bp)	44,578	
	Shortest (bp)	1,321	
	N50 sequence length (bp)	17,267	
	N90 sequence length (bp)	12,269	
Genome assembly statistics			
	Chromosome	Megaplasmid 1	Plasmid 2
Size (bp)	4,376,624	274,256	95,657
GC (%)	63.15	60.71	61.38
Genes	4,064	270	97
rRNAs (5S, 16S, and 23S)	6, 6, and 6	0	0
ncRNA	29	0	0
tRNA	54	0	0
Pseudogenes	327	26	1
CRISPR arrays	2	0	0

^*a*^ CRISPR, clustered regularly interspaced short palindromic repeats.

**Fig 1:**
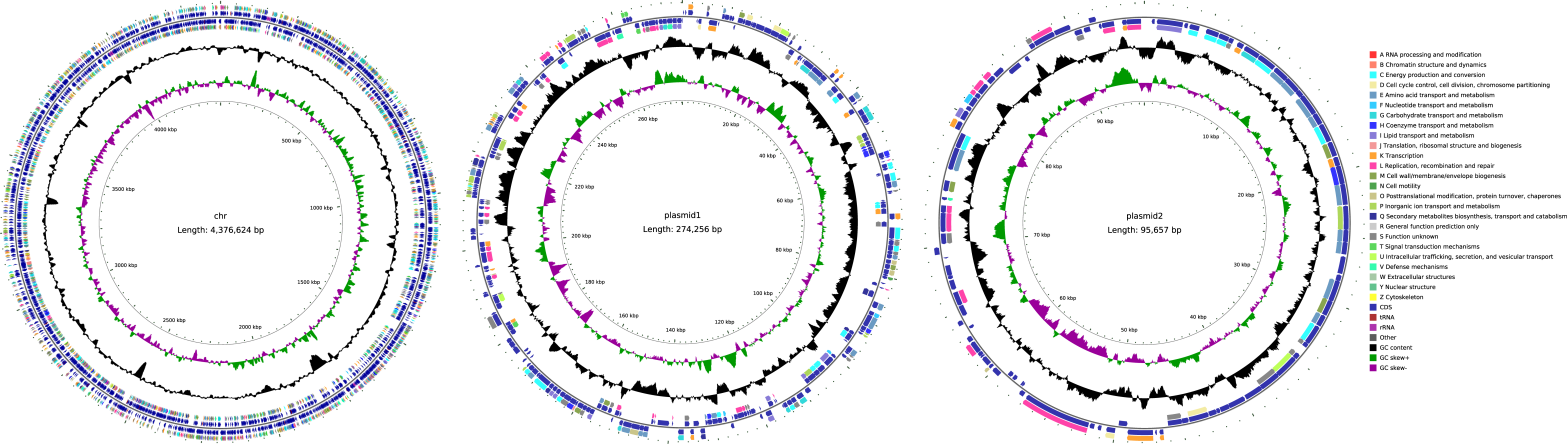
Circular maps of the genome sequences: The *Paenarthrobacter nicotinovorans* FJ15 chromosome, its megaplasmid1, and plasmid2. The maps were generated using circular GenomeViewer (CGView) v2020. Note: plasmid1 is megaplasmid1.

Flye (v2.9.1-b1781) (7) was run with the “--plasmids” option, using the parameters “--pacbio-hifi” and “--genome-size 5m,” resulting in a closed circular sequence (plasmid1) of 274,259 bp. Subsequently, assembly refinement was carried out: the chromosome and plasmid 2 were assembled and polished using Unicycler (v0.5.0; [8]) with default parameters. These sequences were rotated to begin with the replication protein gene using Circlator (v1.5.5) and followed by nine rounds of error correction with Pilon (v1.24; [9]) using the Illumina reads. All final sequences are closed circular assemblies. The circular genome maps are presented in Fig. 1.

Prediction of gene and nc RNA was performed by GeneMark (v4.32; 10), tRNAscan-SE (v2.0.10; 11), and Barrnap v0.9 (12). Default parameters were used for all software unless otherwise specified. Information on the PacBio and Illumina reads is summarized in Table 1. Using FastANI (v1.33; 13), we identified the closest relative of FJ15 as *P. nicotinovorans* strain GCF_021919345.1, with an average nucleotide identity of 98.69%.

The most important finding is that compared to the megaplasmid pAO1 genome (167 kbp) of ATCC49919 (14), the megaplasmid1 genome of *P. nicotinovorans* FJ15 is significantly larger. Based on the Swiss-Prot database, the *hdnoR* gene (a key regulatory factor for nicotine degradation) is located on megaplasmid 1 with 100% similarity (15). These data provide a scientific basis for helping to understand its nicotine catabolic pathway.

## Data Availability

The complete genome sequence of Paenarthrobacter nicotinovorans FJ15 has been deposited at DDBJ/ENA/GenBank under the accession GCA_050632375.1. The associated raw sequencing data (Illumina and PacBio) are available in the SRA under accessions SRR35865469 and SRR36804894, which is part of BioProject PRJNA1264549 and BioSample SAMN48564267.
